# Characterizing tongue deformations during mastication using changes in planar components of three-dimensional angles

**DOI:** 10.1098/rstb.2022.0555

**Published:** 2023-12-04

**Authors:** Rachel A. Olson, Stephane J. Montuelle, Susan H. Williams

**Affiliations:** ^1^ Department of Biology, University of Akron, 302 Buchtel Commons, Akron, OH 44325, USA; ^2^ Department of Biomedical Sciences, Ohio University Heritage College of Osteopathic Medicine, Warrensville Heights, OH 44122, USA; ^3^ Department of Biomedical Sciences, Ohio University Heritage College of Osteopathic Medicine, 228 Irvine Hall, Athens, OH 45701, USA

**Keywords:** chewing, pig, transverse, sagittal, bend, arch

## Abstract

Understanding of tongue deformations during mammalian mastication is limited, but has benefited from recent developments in multiplanar imaging technology. Here, we demonstrate how a standardized radiopaque marker implant configuration and biplanar fluoroscopy can quantify three-dimensional shape changes during chewing in pigs. Transverse and sagittal components of the three-dimensional angle between markers enable characterizing deformations in anatomically relevant directions. The transverse component illustrates bending to the left or to the right, which can occur symmetrically or asymmetrically, the latter sometimes indicating regional widening. The sagittal component reflects ‘arching’ or convex deformations in the dorsoventral dimension symmetrically or asymmetrically, the latter characteristic of twisting. Trends are detected in both the transverse and sagittal planes, and combinations thereof, to modify tongue shape in complex deformations. Both the transverse and sagittal components were also measured at key jaw and tongue positions, demonstrating variability particularly with respect to maximum and minimum gape. This highlights the fact that unlike tongue position, tongue deformations are more independent of jaw position, likely in response to the ever-changing bolus shape and position. From a methodological perspective, our study showcases advantages of a repeatable three-marker implant configuration suitable for animals of different sizes and highlights considerations for different implant patterns.

This article is part of the theme issue ‘Food processing and nutritional assimilation in animals’.

## 1. Introduction

Mandibular kinematics and associated occlusal relations have long been a significant focus of studies of mammalian mastication. However, jaw movements do not occur in isolation. As emphasized by Hiiemae and Crompton [1, p. 263], ‘It is only recently that experimental studies have shown that the tongue and the hyoid apparatus, as well as the soft palate, have a pivotal role in the feeding process and, indeed, may have the primary role…’ Because the tongue lacks a joint and has no internal bony structure, it is not subjected to the typical limits imposed on virtually all other skeletal muscles. As a muscular hydrostat, the mammalian tongue primarily consists of a complex web of interdigitating muscle fascicles running in many directions. Other components include connective tissue and fat [2–4]. Muscle fibres within the tongue show regional differences in their contribution to the tongue's anatomical structure [2]. Accordingly, the movements and deformations of the tongue are significantly more complex than just lengthening and shortening and protraction and retraction [5–7]. It can change its shape in virtually any dimension resulting in side-to-side or sagittal bending or twisting.

Tongue movements and deformations are particularly difficult to characterize, even with fluoroscopy, during routine oral behaviours, such as drinking, speaking and feeding. Not only do these movements and deformations occur within the oral cavity behind the more radiodense dentition, tongue shape constantly changes during these behaviours. Video-fluoroscopy and MRI studies have captured patterns of fibre orientations and general tongue shape, but at relatively low spatial and temporal resolution, and mostly during liquid swallows and speech (e.g. [8–11]). These behaviours use more or less symmetrical changes in tongue position and shape. Chewing, on the other hand, presents a more complex challenge for understanding tongue biomechanics because its deformations tend to be bilaterally asymmetrical. Chewing is typically, but not always, a ‘sided’ behaviour with food being shifted within the oral cavity by the tongue, which works in conjunction with the cheek to manage bolus placement on the teeth [12,13]. Most studies of tongue biomechanics have focused primarily on overall tongue movements, and their coordination with the jaw, with reference to deformations being inferred from bolus positioning rather than through direct assessment or analysis of tissue deformation (e.g. [13–15]).

To overcome this challenge, Abd-el-Malek [5] visually inspected the tongue in patients with missing teeth as they chewed to describe tongue deformations during critical parts of a feeding sequence. This study highlights tongue twisting as an important component of bolus placement (termed the ‘throwing stage’) on the working-side (i.e. chewing side) occlusal surfaces for chewing. More recently, Feilich *et al.* [6] used X-Ray Reconstruction of Moving Morphology (XROMM; [16]) with soft-tissue markers in the tongue of macaques to show that during this ‘throwing stage’, the tongue rolls towards the working side, with the tongue tip towards the balancing side, and that there is sagittal flexion of the tongue, as illustrated by Abd-el-Malek [5]. Our own XROMM studies of pig chewing [7,17,18] have shown that protrusion–retraction and regional anteroposterior and mediolateral linear deformations occur rhythmically in coordination with jaw opening and closing. In addition to confirming tongue–jaw coordination during chewing, these studies highlight how tongue positioning, and multiple simultaneous deformations across different tongue regions are necessary for successful food management and processing.

Understanding the myriad potential movements and deformations of the tongue can be enhanced by marker-based approaches, with careful consideration of marker placement. For example, movements such as protrusion and retraction (measured as the location of a single marker) may occur in a single anatomical dimension (e.g. pure protraction) or across multiple planes (e.g. protraction with superolateral movement). In addition, measuring linear distance between marker pairs quantifies regional changes in a single dimension, such as changes in length or width [7,18]. As described above, the tongue also undergoes complex three-dimensional deformations, which must be measured between sets of three or more markers [6]. The goal of the present study is to highlight how a relatively simple standardized marker arrangement, applicable to a variety of animals of different sizes, can be used to characterize different levels of complexity in tongue deformation. In particular, we demonstrate the potential shape changes observed with markers implanted parallel to the rostrocaudal axis in the body of a pig tongue during chewing. We also highlight the common timing of the deformations within the gape cycle and interpret their potential functional significance with respect to chewing. Finally, we discuss future considerations for marker implant locations to capture different functional deformations.

## 2. Methods

### (a) Standard XROMM methods

**Figure 1.**
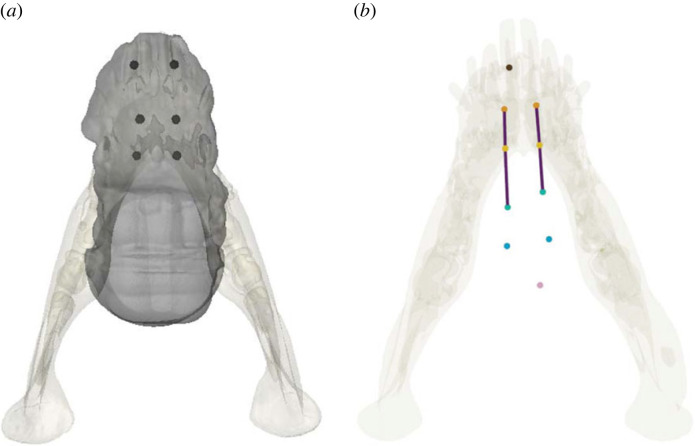
Superior view of a reconstructed jaw showing marker implant locations within the body of the pig tongue. Markers, indicated by coloured circles, are implanted parallel to the *x* or rostrocaudal axis. (*a*) Location of markers relative to the body of the tongue (adapted from Olson *et al.* [18]) and (*b*) lines connect sequential sets of the three left and right markers referenced in this study to infer tongue deformations. These markers were chosen as this is the most freely mobile part of the tongue.

Methods follow our previous studies on chewing in pigs in which markers embedded in the tongue are used in conjunction with bone markers [7,17,18]. Briefly, 5–7 1.6 mm spherical markers (Bal-Tec, Los Angeles, CA, USA) were implanted in each rigid body (skull and jaw) under surgical anesthesia using isoflurane in two female juvenile pigs (*Sus scrofa;* pigs 20 and 21). Additional markers were implanted throughout the anterior two-thirds of the tongue, rostral to the terminal sulcus, using a sterile needle and wire as a plunger. The most anterior marker was placed near the tongue tip in the midline, the most posterior just anterior to the terminal sulcus in the midline, and four right–left pairs were inserted roughly equally spaced from each other midway between the midline of the tongue and the lateral border. All markers were inserted to approximately half of the tongue thickness at their respective locations. As the needle left a tract in the tongue until healed and markers scarred into place, some shifting of markers was observed. Tongue marker locations are shown in figure 1. Following marker implant, animals were CT scanned on a GE Lightspeed Ultra CT scanner at The Ohio State University College of Veterinary Medicine (Columbus, OH, USA). Postmortem CT scans were performed on a Philips Brilliance 64 scanner at Holzer Clinic (Athens, OH, USA). Animals were recorded while chewing 1 cm × 1 cm × 1 cm apple pieces freely available in a bowl. A total of 40 cycles (24 left, 16 right) are analysed here. The classic XROMM workflow [16,19] was followed in XMALab version 1.5.0 to produce the animations in Maya (Autodesk Inc., San Rafael, CA, USA) and extract the corresponding movement data. Additional details on the specifics of the study, including calibration details and precision threshold values to detect real biological motion for the subjects that were included in the present study, are available in previous published reports [17,18]. All aspects of this research complied with the ARRIVE (Animal Research: Reporting of *in vivo* Experiments) guidelines, and were conducted with approval of the Ohio University Institutional Animal Care and Use Committee (protocol #12-U-009).

### (b) Three-dimensional deformations

**Figure 2.**
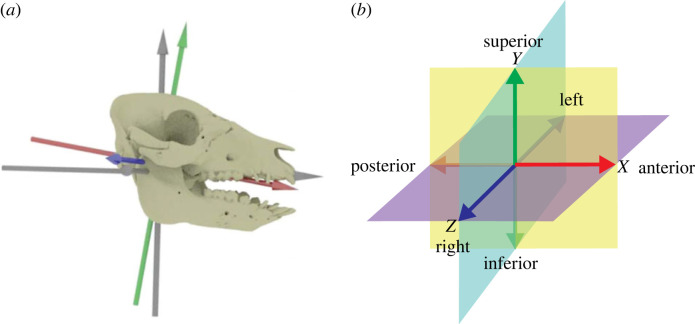
(*a*) Anatomical coordinate system used for movements of tongue markers (coloured) relative to the jaw versus the joint coordinate system (grey) used to calculate movements of the jaw relative to the skull. (*b*) Anatomical planes relative to the standard XROMM joint coordinate system. Red, anteroposterior *x*-axis; green, dorsoventral *y*-axis; blue, mediolateral *z*-axis; yellow, sagittal (*XY*) plane; purple, transverse (*XZ*) plane; teal, frontal (*YZ*) plane.

To analyze three-dimensional deformations, the three-dimensional angle between three consecutive soft-tissue markers, implanted in the right and left sides of the tongue (figure 1), and its corresponding two-dimensional components along functionally relevant planes were calculated and adjusted to the location of the markers during a CT scan with the tongue in neutral position. Following typical joint coordinate system (JCS) orientation for feeding XROMM research, the coordinate system was oriented with an anteroposterior *x*-axis, superoinferior *y*-axis, and a mediolateral *z*-axis oriented through the centre of joint rotation of the mandibular condyles and along the hard palate. This axis was parented to the jaw object, this ensures tongue marker positions track jaw position. The resulting planes are the sagittal (*XY*), transverse (*XZ*) and frontal (*YZ*) planes (figure 2).

To better understand the patterns of tongue deformations, we measured changes in the angle components (i.e. transverse and sagittal plane) at various timepoints within the gape cycle: at maximum gape (i.e. start of the gape cycle), minimum gape, maximum tongue retraction, and maximum tongue protraction. Mean (± standard deviation, s.d.) transverse and sagittal components of the three-dimensional angle, relative to neutral position from the CT scan, were determined for both the right and left sets of markers of selected chews that could be kinematically identified as left or right chews according to jaw movements (see [16,17]). Angle values were adjusted such that rest position was 0° in both transverse and sagittal planes, indicating the tongue is straight and flat. For the transverse component, positive values indicate bending to the left, and negative values illustrate bending to the right. For the sagittal component, positive values reflect arching or a convex shape of the dorsal surface of the tongue, and negative values indicate cupping or a concave shape of the dorsal tongue surface.

## 3. Results

### (a) Tongue deformations during chewing

**Figure 3.**
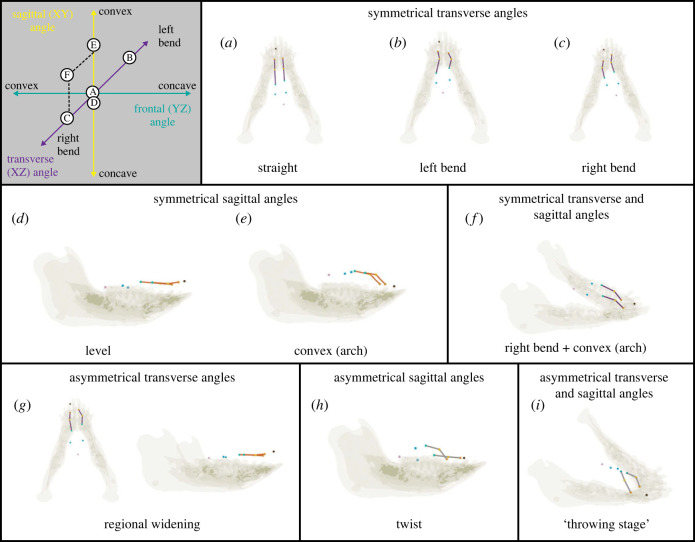
Deformations of the tongue during a chewing sequence (pig 21, apple) inferred from the selected markers in figure 1. The reference system (grey panel) shows the three planar components of the three-dimensional angle formed by three markers (with respect to the anatomical planes in figure 2). The double-arrowhead lines represent hypothetical continua of planar components formed by the markers as the tongue deforms from rest position. Locations of the letters A–F along the axes are representative of the planar components of the three-dimensional angle of the right marker set for the symmetrical deformations shown in the top two rows. G–I are asymmetrical deformations and for simplicity, are not shown in the grey panel. Timepoints where deformations A–I occur are indicated on figure 4. Coloured lines joining right and left marker sets indicate the plane of deformation (purple, transverse plane; orange, sagittal plane; grey, multiple planes).

Several primary patterns of tongue shape changes in the sagittal and transverse planes (figure 3) were extracted from the angle of the three consecutive markers embedded in the mid-region of the tongue (figure 1). This region was selected as it was the most freely mobile portion of the tongue. Deformations from resting position in the transverse plane are reflected as positive (left bend: figure 3*b*) and negative (right bend: figure 3*c*) angles made by the three markers when the anterior and posterior markers are offset in the same direction from the central marker. Bending was commonly observed throughout the gape cycle. Changes in the direction of bending by necessity means that at certain points during its shape change, the tongue passes through an instantaneous neutral, or straight, position but this was not a primary ‘posture’ during chewing (figure 3*a*).

**Figure 4.**
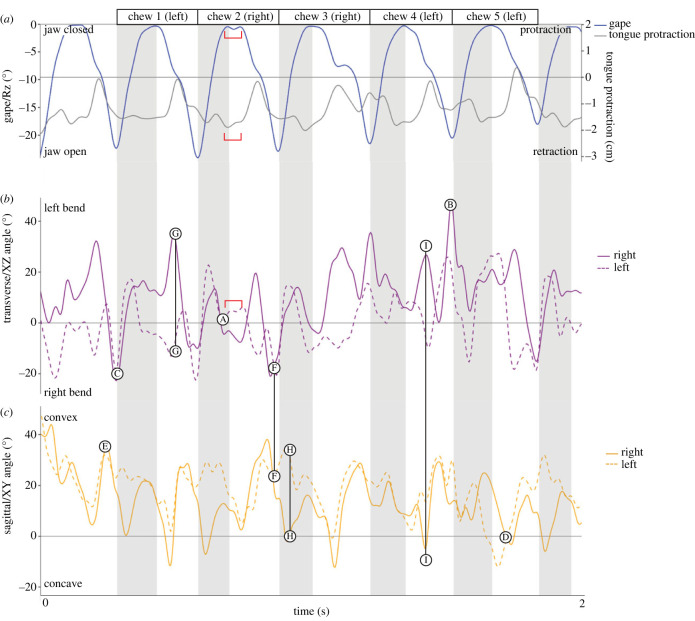
Tongue deformations from resting position relative to the gape cycle (*a*) in the transverse (*b*) and sagittal (*c*) planes during sequential chewing cycles. (*a*) Blue (left axis), Rz (gape) used to determine cycles; green (right axis), Ry (yaw) used to side cycles. Cycle side is indicated at the top. Grey shading, jaw closing; white shading, jaw opening. Solid and dashed waves represent the right and left marker sets, respectively. Circled letters correspond to the letters in figure 3. Connecting lines demonstrate differences in right–left marker sets (G,H) or between planes (F,I). In (*a*) and (*b*), the red brackets highlight the occlusal phase for one cycle, when the tongue is retracted and slightly asymmetrically bent in the transverse plane.

In the sagittal plane, ‘arching’ or convex deformations were also easily observed (figure 3*f*; increasing angle) as well as level to slightly ‘dipped’ or concave shapes (figure 3*e*; decreasing angle). These deformations could be similar bilaterally (figure 3*a–c, e–f*) or they could differ in magnitude and/or direction (figure 3*g*). Finally, deformations were routinely observed simultaneously in both planes (figure 4).

### (b) Tongue deformations relative to jaw kinematics during chewing

These three-dimensional shape changes occur continuously at different times during the gape cycle (see electronic supplementary material, video S1). The first major pattern we observed is coordinated mediolateral bending between right and left marker sets in the same direction. The peak magnitude bend occurs consistently during late opening, near maximum gape (figure 4: timepoints B,C). The middle marker of the set may be oriented towards the balancing side (figure 4: timepoint B) or the working side (figure 4: timepoint C). Lower magnitudes of transverse bending (approximately half of the maximum observed angles) occur during closing, sometimes straightening (figure 4: timepoint A) very briefly when switching bending directions. Opposite bending directions between right and left marker sets are regularly observed during mid-opening (figure 4: timepoint G) as a result of widening of the tongue between the central markers and narrowing of the anterior and posterior markers in this plane. This almost always occurred during jaw opening.

**Figure 5.**
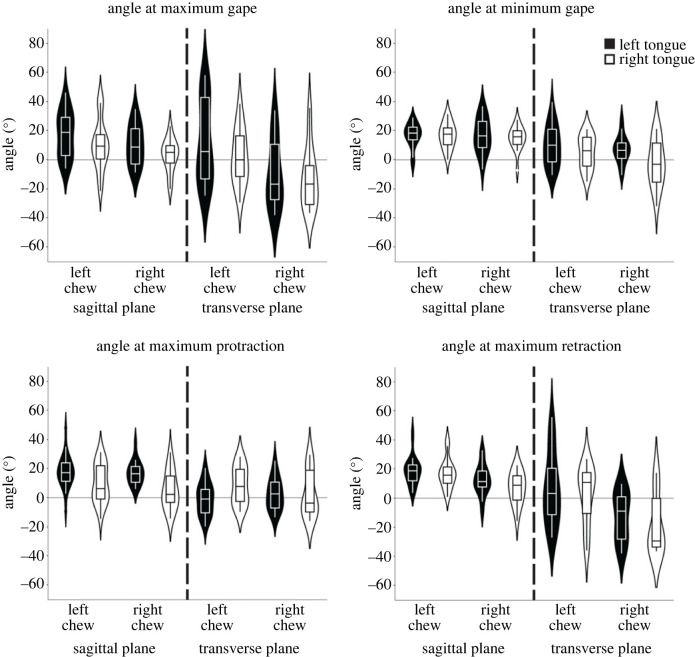
Angle measurements of right and left marker sets in the sagittal and transverse planes during key timepoints: maximum gape (cycle start), minimum gape, maximum tongue protraction, and maximum tongue retraction. Both individuals are pooled for left chews (*N* = 24) and right chews (*N* = 16). Corresponding mean and standard deviations relative to rest position are reported in electronic supplementary material, table S2.

In the sagittal plane, three main patterns are observed. The tongue has a positive sagittal bend angle, or arch, bilaterally during mid-to-late opening (figure 4: timepoint E). A smaller bilateral positive arch is sometimes observed during closing. A flattened tongue in the sagittal plane (or even a slight negative angle) is observed during early-to-mid opening (figure 4: timepoint D). Finally, non-symmetrical bends are observed commonly during closing (figure 4: timepoint H), but do not occur in every cycle.

Note these deformations do not have to occur in isolation as illustrated in figure 3*f–i* where deformations are observed in both the transverse and sagittal planes for the same marker set. This corresponds with figure 4*b,c* where deformations in both planes are constantly occurring simultaneously.

### (c) Tongue deformations in relation to cycle and tongue parameters

Because event-based understanding of masticatory dynamics is entrenched in the descriptive literature on mammalian chewing, we evaluated tongue ‘posture’ at four key events during the gape cycle: start of the gape cycle (maximum gape), minimum gape, maximum tongue protrusion, and maximum tongue retraction. These data are presented in figure 5 and electronic supplementary material, table S2 summarizes the mean transverse and sagittal angular components, relative to rest position, of tongue deformation observed at these events. Tongue configurations at the different timepoints are highly variable in both planes, revealing that tongue deformations are not constrained by jaw position. In particular, the highest variation seems to be observed in the transverse plane at maximum gape, which corresponds to the end of jaw opening and the start of jaw closing. However, in 83% of the cycles (20/24 left chews and 13/16 right chews; electronic supplementary material, table S1), both sides of the tongue are bent in the same transverse direction at maximum gape. In the sagittal plane, the tongue at maximum gape has a slightly greater convex arch on the left side than on the right side for both left- and right-sided chews.

At minimum gape, we observed the mean values for the left and right chews to show a small positive change (except right chews/right side) in the transverse angle, indicating a bend to the left when teeth are maximally occluded. However, there is significant variation at the level of individual chews with some cycles showing bending towards the right, regional narrowing or regional widening. In the sagittal plane, the tongue maintains a bilateral convex arch for left and right chews.

At maximum tongue protraction, which occurs approximately in the middle of jaw opening, the low transverse angle defined by the left and right markers indicates a more or less straight tongue. At this time, the left side is more convexly arched in the sagittal plane than the right side, regardless of the chewing side. In other words, the right side of the tongue is flatter than the left side when the tongue is maximally protracted, as in figure 3*h*.

At maximum tongue retraction, shortly before the jaw is fully closed, the transverse angle change is positive for the right and left markers during left chews but negative during right chews, indicating that the tongue bends towards the working side when it is retracted. While there is significant variation at the cycle level, left and right sides tend to bend in the same direction (see electronic supplementary material, table S1). In the sagittal plane, the two sides of the tongue are similarly deformed, convexly arched in this case, when the tongue is maximally retracted.

## 4. Discussion

With a relatively simple marker configuration consisting of three rostrocaudally arranged markers on each side of the tongue, we are able to demonstrate some general patterns in tongue deformations during chewing from qualitative observations. Symmetrical bending in the transverse plane (e.g. towards the left or the right) was commonly observed during late jaw opening, just before maximum gape (figure 4*b*: timepoints B,C) with a reversal of the direction of symmetrical bending towards the other side during jaw closing. This pattern appeared regardless of the chewing side. This is notably different than that observed in humans. Mioche *et al*. [13] demonstrated that tongue-pushing towards the working side to move or hold food on the occlusal surfaces was correlated with definitive unilateral chewing on that side. Thus while tongue-jaw coordination is preserved at the gape-cycle level, the relationship between tongue transverse deformation to chewing side is less constrained in pigs. This difference may reflect the fact that pigs tend to occlude on both sides simultaneously because they are anisognathous [20], and food is likely being manoeuvered between both sides of occluding teeth despite there being a true kinematic working and balancing side as evidenced by jaw yaw [16,21].

Low-magnitude bends or a more or less straight tongue in the transverse plane were observed as a transitional state between the direction of transverse bending. There can be more prolonged periods of low-magnitude symmetrical or asymmetrical bending occurring as the jaw was held together during the occlusal phase, when the tongue is most retracted ([18]; see red brackets in figure 4*a,b*). This likely also helps stabilize the bolus on the occlusal surface, but more importantly, protects the tongue from damage and injuries by the teeth.

Bilateral incongruence in transverse bending was consistently observed mid-opening (figure 4*b*: timepoint G). In these cases, the right marker set reflected a left bend while the left marker set reflected a right bend, such that the middle markers of each set were in a more lateral position than the anterior and posterior markers (i.e. widening in the transverse plane here as the tongue is level in the sagittal plane; figure 3*g*). This roughly corresponds with previous work in this individual, where the timing of maximum width for the middle marker (pig 21, region 2 in [18]) is offset from the timing of the more anterior and posterior marker pairs [18]. We hypothesize this may be a localized particle collection or may be a stage of twisting to help with transport and reposition the bolus.

Sagittal angles showed consistent timing relative to the gape cycle. The maximum bilaterally positive (convex) angle, or arch, was observed during mid-to-late opening (figure 4*c*: timepoint F) whereas the minimum bilateral arch (flat to slightly concave shape) was observed during early-to-mid opening (figure 4*c*: timepoint D). This flat or slightly concave shaped deformation is likely contributing to the formation of a cupped tongue to aid in the collection of particles in preparation for the next powerstroke. The addition of a midline marker to allow for analysis of the frontal angle would confirm this hypothesis. This would align with the ‘preparatory stage’ of Abd-el-Malek [5], where the tongue has a trough-like shape for the collection of particles. This is immediately followed by a quick transition to the large positive arch (long axis flexion of Feilich *et al.* [6] during late opening to position the bolus along the toothrow for the powerstroke.

Differences in sagittal angles between right and left marker sets are often, but not always, observed during closing. Visually, this shape suggests an extreme twisting of the tongue, which is thought to be the main mechanism for repositioning the bolus. However, our observations of bilaterally asymmetrical sagittal bending again does not conform to when side-switches occur as one would expect. Increased resolution in quantifying or measuring the bolus and food particles positions within the oral cavity is necessary to understand the function of this type of deformation.

Finally, it is important to remember that deformations are three-dimensional, often occurring across multiple planes simultaneously. For example, the timing of the bilateral maximal transverse angle is quite similar to when the maximum sagittal angle occurs (figure 4: timepoint F). These complex, coordination deformations work together to handle the bolus during mastication. The ‘throwing stage’ of Abd-el-Malek [5] observed twisting of the tongue to position the bolus on the occlusal surface. Specifically, the middle of the tongue twists so that the dorsal surface of the tongue faces the working side. Experimental work on macaques by Feilich *et al.* [6] demonstrates a similar type of deformation occurring during jaw closing. In this study, a transverse bend combined with a convex arch is observed at this moment. Although this shape is often observed during chewing here (figure 3*i*; electronic supplementary material, video S1), it is usually observed during opening or at the transition from opening to closing (figure 4: timepoint I). Additional data including the marker in the tip of the tongue would help clarify the shape changes observed here.

Previous work [7,17,18,22,23] has shown that anterior and posterior tongue widening and lengthening are more or less congruent with each other and occur consistently in coordination with tongue protrusion and retraction and/or jaw opening and closing. However, particularly for jaw closing, instantaneous jaw or tongue position as shown in figure 5, and corresponding tongue shape may not be as clearly linked. This reflects the constant deformations that occur during chewing to handle the bolus.

Finally, the left and right sides of the tongue tend to differ in their pattern of deformation during chewing, reinforcing the asymmetrical nature of this behaviour. Moreover, the complexity of deformation is higher than the anteroposterior length deformations observed by Olson *et al.* [18], with rapidly changing directions of bending occurring several times during the gape cycle versus a more or less consistent increase in length and narrowing of the tongue that occurs from jaw closing to jaw opening. The present study clarifies how linear deformations occur, as length and width changes are absolute distances that disregard orientation in three-dimensional space. For example, in figure 3*g* the tongue is wider in the middle set of right–left markers than between the more anterior and posterior set. This could be a true widening deformation, or it could be that there is similar absolute distance between the anterior and posterior marker sets as well, just occurring out of plane. The sagittal still image of the same frame confirms that this is true widening along the transverse plane. Correspondence of absolute linear distances with planar components in the same dataset increases the depth of data interpretations possible in different scenarios.

## 5. Conclusion

The tongue displays near constant and complex patterns of deformations in the transverse and sagittal planes during chewing in the pig. Contrary to expectations, the working side occlusal surface does not correspond to the direction of tongue deformations. Therefore, chew side and side switches cannot be accurately determined from tongue deformations alone. The magnitude and timing of the deformations are fairly consistent in relation to jaw movements, but are not necessarily predicted by jaw or tongue kinematic events (e.g. maximum gape). Combined, these results reflect that tongue deformations, but not positional changes, are more unconstrained from jaw movements and instead adapt to adjust to an ever-changing bolus.

Although there have been great methodological improvements in the collection and processing of three-dimensional kinematic data [24], further work is necessary to determine the functional significance of many tongue shape changes. Careful selection of soft tissue marker locations is necessary to capture the movements and deformations of interest or to address a specific question. The marker implant pattern used here, which should be suitable for most animals with a fleshy tongue, captures deformation in the sagittal and transverse planes. However, any deformations observed in the frontal plane from these marker sets cannot be resolved. Addition of midline markers between the right–left marker pairs would provide the necessary resolution in this plane to resolve whether left to right arching or cupping occurs. Similar limitations were observed in the implant patterns in macaques [6,25]. Tongue angle (here referred to as arching) was calculated using three consecutive markers. Two markers implanted parallel to the *z*-axis were used to calculate tongue roll, however, this does not allow for determination of the angle in the frontal plane (formation of a rostral–caudal trough). Finally, visualization of the food particles within the oral cavity (e.g. [13]), in particular their movements and orientations relative to each other and the corresponding anatomical structures, would clarify the movements observed here and allow for more-specific hypothesis testing, especially regarding foods of different mechanical properties.

The approach and methods presented can be applied to a myriad of applications for soft tissue research. Much remains to be learned across different model systems, diets, food mechanical properties for questions spanning form–function relationships, evolution and clinical perspectives. Our results are a small representation of the data that can be gleaned from markers implanted in one array within a soft tissue structure. Careful consideration of what data are needed to address questions of interest will aid in developing a marker implant pattern that can best capture complex movements.

## Data Availability

Cycle level angular data for table 1 are available as electronic supplementary material, table S1. Data presented in figure 5 are available in electronic supplementary material, table S2. Code and relevant wave files are available on the XMAPortal in the collection entitled ‘Characterizing tongue deformations during mastication using changes in planar components of three-dimensional angles’. The data are provided in electronic supplementary material [26].
